# Gene expression profiling in the lungs of pigs with different susceptibilities to Glässer's disease

**DOI:** 10.1186/1471-2164-11-455

**Published:** 2010-07-29

**Authors:** Jamie M Wilkinson, Carole A Sargent, Lucina Galina-Pantoja, Alexander W Tucker

**Affiliations:** 1 Department of Veterinary Medicine, University of Cambridge, Madingley Rd, Cambridge, CB3 0ES, UK; 2 Department of Pathology, University of Cambridge, Tennis Court Rd, Cambridge, CB2 1QP, UK; 3 PIC North America, 100 Bluegrass Commons Blvd, Suite 2200, Hendersonville, Nashville, TN, 37075, USA; 4 Department of Agricultural, Food, and Nutritional Science, University of Alberta, 4-23G Agriculture/Forestry Centre, Edmonton, Alberta, T6G 2P5, Canada; 5 Current Address: Pfizer Animal Health, 235 East 42nd Street, New York, NY, USA

## Abstract

**Background:**

*Haemophilus parasuis *is the causative agent of Glässer's disease in pigs. Currently, little is known about the molecular mechanisms that contribute to disease susceptibility. This study used a porcine oligonucleotide microarray to identify genes that were differentially expressed (DE) in the lungs of colostrum-deprived animals previously characterized as being either 'Fully Resistant' (FR) or 'Susceptible' to infection by *H. parasuis *in a bacterial challenge experiment.

**Results:**

Gene expression profiles of 'FR' and 'Susceptible' animals were obtained by the identification of genes that were differentially expressed between each of these groups and mock-inoculated 'Control' animals. At 24 hours post-inoculation, a total of 21 and 58 DE genes were identified in 'FR' and 'Susceptible' animals respectively. At 72 hours, the numbers of genes were 20 and 347 respectively. 'FR' animals at 24 hours exhibited an increased expression of genes encoding extracellular matrix and TGF-β signalling components, possibly indicative of tissue repair following the successful early resolution of infection. The gene expression profile of 'FR' animals at 72 hours supported the hypothesis that higher levels of antibacterial activity were responsible for the 'FR' phenotype, possibly due to an increase in natural immunoglobulin A and decrease in signalling by the immunoregulatory transcription factor peroxisome proliferator-activated receptor gamma (PPAR-γ). The expression profile of 'Susceptible' animals at both time-points was characterized by an imbalance in signalling between pro and anti-inflammatory cytokines and an increased expression of genes involved in biological processes associated with inflammation. These include the pro-inflammatory cytokine genes resistin (*RETN*) and interleukin 1-beta (*IL1B*). At 72 hours, a reduction in the expression of genes involved in antigen presentation by both MHC class I and II molecules was observed, which could have contributed to the inability of 'Susceptible' animals to clear infection.

**Conclusions:**

This study is the first to have identified discrete sets of DE genes in pigs of differing susceptibility to *H. parasuis *infection. Consequently, several candidate genes and pathways for disease resistance or susceptibility phenotypes have been identified. In addition, the findings have shed light on the molecular pathology associated with Glässer's disease.

## Background

Glässer's disease in swine is caused by the bacterium *Haemophilus parasuis*. This bacterium is commonly isolated from the upper respiratory tract of healthy pigs. However, in some animals the bacterium can breach the mucosal epithelium and spread systemically to cause disease, by means that are poorly understood [1]. Glässer's disease is characterized by polyserositis, arthritis and meningitis. It can be fatal and typically affects pigs 6-8 weeks of age. Fifteen serotypes of *H. parasuis *exhibiting varying degrees of virulence can be identified using the 'Kielstein and Rapp-Gabrielson' scheme [2]. However, approximately 30% of field strains are untypable by this method, and there is no absolute correlation between virulence and serotype, an indication of the high heterogeneity of *H. parasuis *isolates [3]. Vaccines against *H. parasuis *are commercially available, but none of the vaccines offer comprehensive protection against all heterologous strains, and are occasionally ineffective against homologous strains as well [4-6]. Consequently, there is a need to investigate other methods of controlling the disease such as improving the disease resistance of pig populations through marker assisted selection.

It is clear that the immune status of the piglet is critically important in determining the outcome of *H. parasuis *infection. Attempts to reproduce Glässer's disease experimentally have only been consistently successful when using animals from specific pathogen free (SPF) herds or colostrum-deprived (CD) piglets [7-10]. These results underscore the importance of antibodies to *H. parasuis *in conferring protection from disease. Indeed, the role of maternal immunity in the protection of neonatal pigs has recently been demonstrated [11,12]. It has been postulated that the vulnerability to infection of animals aged 6-8 weeks is due to a decline in the amount of circulating maternal antibody post-weaning. Pigs that are exposed to *H. parasuis *at an earlier age, and are protected initially by maternal immunity, have sufficient time to develop their own antibody response to the bacterium [1]. There is evidence that the controlled early exposure of pigs to the prevalent farm strain of *H. parasuis *can reduce mortality levels, however there are concerns about exposing young pigs to live, virulent bacteria with this method [13].

Blanco *et al. *observed large differences in susceptibility to Glässer's disease in CD animals inoculated with pathogenic *H. parasuis *under identical conditions, indicating that susceptibility to the disease may also have a genetic component [12]. In a larger scale challenge experiment, the 21 day old CD offspring of 6 different sires were either inoculated with *H. parasuis *or mock-inoculated with saline. Approximately equal numbers of animals were euthanized at 24, 48, and 72 hours post-inoculation and categorized into different disease susceptibility categories based on scores for the presence of bacteria, lesions, and clinical signs associated with the disease [14]. The goal of this current study was to extend the work of Blanco *et al. *[14] by using microarrays to define gene expression profiles of piglets identified in that study as exhibiting low ('Fully Resistant') or high ('Susceptible') susceptibility to Glässer's disease at early (24 hours) and late (72 hours) stages of the challenge experiment. Since all pigs were challenged by intra-tracheal inoculation, the investigation focussed on lung tissue.

Microarrays have recently been employed successfully to identify the molecular pathways involved in the pig response to a number of different microbial pathogens and to identify disease resistance candidate genes [15-18]. In addition to characterizing the host response to *H. parasuis *infection in animals of differing susceptibility, this project aimed to identify the pathways involved in the molecular pathology associated with Glässer's disease.

## Results

### Validation of infection status in the lungs of 'FR', 'Susceptible', and 'Control' pigs

**Figure 1:**
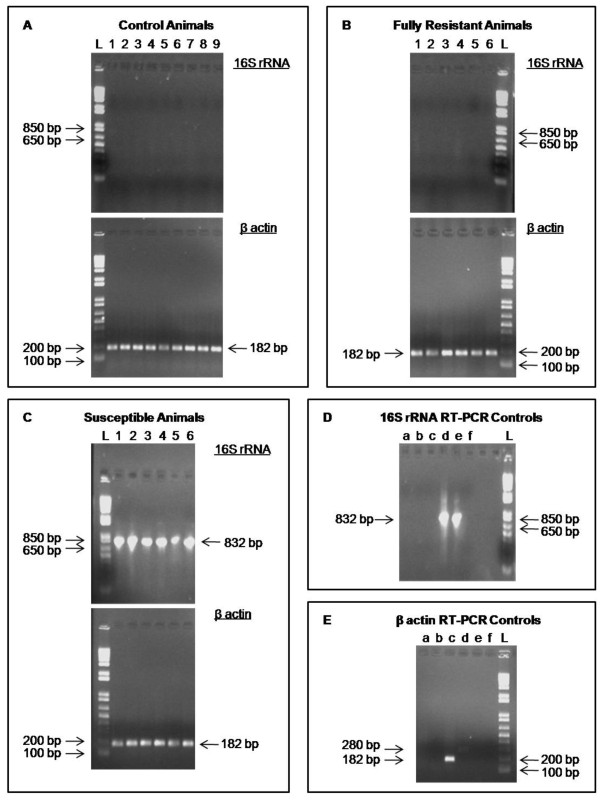
**Validation of *H. parasuis *infection status in 'Fully Resistant', 'Susceptible' and mock-inoculated 'Control' animals**. RT-PCR using *H. parasuis *16 S rRNA and pig β-actin primers on RNA from (A) mock-inoculated 'Control' animals, (B) 'Fully Resistant' animals and (C) 'Susceptible' animals. L denotes the DNA ladder lane. The number above each lane denotes the animal tested. For example lane 1 in panel A denotes animal C1; lane 5 in panel C denotes animal S5. The 16 S rRNA amplicon is 821 bp in size when either genomic DNA or cDNA is used as template. The β-actin amplicon is 182 bp in size when cDNA is used as template and 280 bp when genomic DNA is used. The sizes of molecular standards in the DNA ladder are indicated for comparison with amplicon sizes. PCR and RT-PCR controls for (D) 16 S rRNA and (E) β-actin reactions are also provided. The lower case letter above each lane denotes a specific control. For panel D: (a) RT-PCR on uninfected pig RNA. (b) No reverse transcriptase RT-PCR on uninfected pig DNA. (c) PCR on pig genomic DNA. (d) RT-PCR on *H. parasuis *RNA. (e) PCR on *H. parasuis *genomic DNA. (f) PCR negative control. For panel E: (a) RT-PCR on *H. parasuis *RNA. (b) PCR on *H. parasuis *genomic DNA. (c) RT-PCR on uninfected pig RNA. (d) PCR on pig genomic DNA. (e) No reverse transcriptase RT-PCR on uninfected pig RNA. (f) PCR negative control. Results shown are from one of two independent replicate RT-PCR experiments.

In the study of Blanco *et al. *[14], pigs classified as 'Fully Resistant' had no *H. parasuis *present at internal sites typically affected by the disease, including the lung, as determined by bacterial culture or PCR. In addition, only minor clinical signs or limited pathology at a small number of sites were permitted. In contrast, 'Susceptible' animals exhibited multiple lesions and clinical signs of Glässer's disease, and bacteria were isolated from several internal sites by both culture and PCR. Prior to carrying out microarray experiments, the absence of detectable *H. parasuis *in the lungs of each of the 'FR' and 'Control' animals, and its presence in 'Susceptible' animals, was confirmed by RT-PCR using primers for *H. parasuis *16 S rRNA [19]. A positive control RT-PCR performed using pig β-actin primers confirmed that the failure to detect 16 S rRNA transcripts in the 'FR' and 'Control' animals was not due to a failure of reverse-transcription (Figure 1). The sensitivity of the RT-PCR test was estimated to be 0.5 bacterial cells using a dilution series of *H. parasuis *RNA spiked into mock-inoculated 'Control' pig lung total RNA carried out in triplicate (data not shown). This was an improvement on the sensitivity of the original PCR test (estimated to be 102 bacterial cells), and strengthened the case for 'FR' animals having no viable *H. parasuis *in lung tissue. The limit of sensitivity of this test is consistent with other RT-PCR tests for microbial species [20,21].

### Overview of gene expression in 'FR' and 'Susceptible' pigs

Two 'FR' and 'Susceptible' individuals at 24 hours, and 4 'FR' and 'Susceptible' individuals at 72 hours post-inoculation, were chosen for microarray experiments. The 'FR' and 'Susceptible' animal groups were matched for sire. Gene expression in the lungs of individual 'FR' or 'Susceptible' individuals was compared to that in a reference RNA made from 3 or 6 mock-inoculated, sire-matched 'Control' animals at the 24 and 72 hour time-points respectively. It was not possible to use multiple 'Control' animals for the same sire and time-point in the pools as only one animal from each sire at each time-point was designated as a 'Control' animal in the original challenge experiments. Therefore pools were made from samples from 'Control' animals of the same sire but a mixture of time-points (24, 48, and 72 hours). A list of the animals used is provided in Table 1. In this design the reference samples are of biological interest and therefore direct comparisons of 'FR' and 'Control' animals, and 'Susceptible' and 'Control' animals; and the indirect comparison of 'FR' and 'Susceptible' animals were made during analysis. At each time-point (24 and 72 hours post inoculation), 4 comparisons of differential gene expression were carried out: 'Fully Resistant' v 'Control' (FR v C), 'Susceptible' v 'Control' (S v C), 'Fully Resistant' v 'Susceptible' (FR v S), and 'Susceptible' v 'Fully Resistant' (S v FR). The last two comparisons are actually from the same analysis; the expression ratios calculated for each gene are the inverse of each other, as the values are dependent on whether the ratio is expressed in terms of the 'Fully Resistant' or 'Susceptible' group. The raw and normalized microarray data used for these comparisons have been submitted to the Gene Expression Omnibus (GEO) database (series entry GSE19126).

**Table 1:** Animals used for microarray experiments

Animal	Sire	Time point (hrs)	Susceptibility Classification
			
24 hour time-point microarray experiment			
			
R5	H78	24	Fully Resistant
R6	H78	24	Fully Resistant
S5	H78	24	Fully Susceptible
S6	H78	24	Fully Susceptible
C7	H78	24	Control
C8	H78	48	Control
C9	H78	72	Control
			
72 hour time-point microarray experiment			
			
R1	H92	72	Fully Resistant
R2	H92	72	Fully Resistant
R3	H77	72	Fully Resistant
R4	H77	72	Fully Resistant
S1	H92	72	Fully Susceptible
S2	H92	72	Fully Susceptible
S3	H77	72	Less Susceptible
S4	H77	72	Less Susceptible
C1	H92	24	Control
C2	H92	48	Control
C3	H92	72	Control
C4	H77	24	Control
C5	H77	48	Control
C6	H77	72	Control

The number of DE genes identified in each of these comparisons is shown in Table 2, while complete gene lists for each of the comparisons are provided in additional files 1 and 2. At 24 hours, roughly similar numbers of DE genes were identified in the FR v C and FR v S (or S v FR) comparisons, 95 and 109 respectively, while a slightly larger number of DE genes, 149, were identified in the S v C comparison. At 72 hours, only 41 genes were differentially expressed in the FR v C comparison. In contrast, a total of 557 and 602 DE genes were found in the S v C and FR v S (or S v FR) comparisons respectively.

**Table 2:** Number of differentially expressed (DE) genes across all microarray comparisons

Comparison	Gene expression difference	No. of DE genes at 24 hours	No. of DE genes at 72 hours
Fully Resistant v Control (FR v C)	More highly expressed in FR than C	46	6
	Less highly expressed in FR than C	49	35
	**Total number of DE genes**	**95**	**41**
Susceptible v Control (S v C)	More highly expressed in S than C	81	191
	Less highly expressed in S than C	68	366
	**Total number of DE genes**	**149**	**557**
Fully Resistant v Susceptible or Susceptible v Fully Resistant (FR v S or S v FR)	More highly expressed in FR than S or less highly expressed in S than FR	64	337
	Less highly expressed in FR than S or more highly expressed in S than FR	45	265
	**Total number of DE genes**	**109**	**602**

### Expression profiles of 'FR' and 'Susceptible' animals

The lists of DE genes generated by microarray analyses were subsequently used to identify the subset of genes that were differentially expressed in 'Fully Resistant' animals compared to both 'Control' and 'Susceptible' (i.e. differentially expressed in the FR v C and FR v S comparisons), and 'Susceptible' animals compared to both 'Control' and 'Fully Resistant' (i.e. differentially expressed in the S v C and S v FR comparisons) at both 24 and 72 hours post inoculation. These genes defined the expression profiles of 'Fully Resistant' and 'Susceptible' animals, and were deemed to be of particular interest for their potential to be differently regulated in response to infection in 'Fully Resistant' and 'Susceptible' animals. The shorthand convention X/Y is used to denote a gene that is differentially expressed between X and Y. Expression of DE genes is described as being either higher or lower in X/Y e.g. higher in FR/C and FR/S indicates a gene whose expression is higher in 'FR' animals compared to both 'Control' and 'Susceptible' animals. Complete lists of the DE genes in these expression profiles are provided in additional files 3 and 4, and summarized in Tables 3, 4, 5, 6, and 7. The terms 'upregulated' and 'downregulated' are avoided because the use of tissue samples precluded the ability to distinguish between expression differences caused by enhancement or repression of transcription or alterations in the proportions of different cell types in the sample. Gene Ontology (GO) terms that were statistically over-represented among genes annotated in these lists were assumed to be of potential functional significance.

**Table 3:** Differentially expressed (DE) genes in the expression profile of 'Fully Resistant' animals 24 hours post-inoculation

Genes grouped by function	Genbank human RefSeq ID	FR v C fold change	FR v S fold change
			
**More highly expressed in 'Fully Resistant' animals**			
			
Extracellular matrix components			
			
**Tenascin C (*TNC*)**	NM_002160	+2.30	+4.20
**TGF-β-induced (*TGFBI*)**	NM_000358	+2.20	+4.69
**Latent TGF-β binding protein 4 (*LTBP4*)**	NM_003573	+2.07	+2.41
**Collagen, type I, alpha 2 (*COL1A2*)**	NM_000089	+2.03	+2.57
**EGF-containing fibulin-like extracellular matrix protein 2 (*EFEMP2*)**	NM_016938	+1.95	+2.01
**Collagen, type III, alpha 1 (*COL3A1*)**	NM_000090	+1.85	+2.43
**Collagen, type XV, alpha 1 (*COL15A1*)**	NM_001855	+1.84	+2.46
**Collagen, type VI, alpha 3 (*COL6A3*)**	NM_057167	+1.77	+2.33
			
Other			
			
X (inactive)-specific transcript (*XIST*)	NR_001564	+2.89	+8.06
Slit homolog 3 (*SLIT3*)	NM_003062	+2.06	+2.57
Prolylcarboxypeptidase (*PRCP*)	NM_005040	+1.89	+2.60
Coiled-coil domain containing 80 (*CCDC80*)	NM_199512	+1.83	+2.43
Oligo SS00010557 (no significant hit to known gene)	NONE	+1.78	+3.36
			
**Less highly expressed in 'Fully Resistant' animals**			
			
Retinoid-binding			
			
**Retinol binding protein 4 (*RBP4*)**	NM_006744	-2.51	-6.54
**Apolipoprotein D (*APOD*)**	NM_001647	-1.80	-2.43
			
Heat shock proteins			
			
**DnaJ, subfamily B, member 1 (*DNAJB1*)**	NM_006145	-2.03	-3.51
**Heat shock 70kDa protein 6 (*HSPA6*)**	NM_002155	-2.20	-3.32
			
Lipid mobilization and metabolism			
			
Lipoprotein Lipase (*LPL*)	NM_000237	-1.92	-5.05
			
Other			
Oligo SS00011159 (no significant hit to known gene)	NONE	-1.77	-3.58
Transcobalamin I (*TCN1*)	NM_001062	-1.82	-4.63
DEAD box polypeptide 3, X-linked (*DDX3X*)	NM_001356	-2.22	-2.36

A list of all genes that were differentially expressed in both the 'FR v C' and 'FR v S' comparisons 24 hours post-inoculation. Gene names were assigned on the basis of the best sequence match to a human gene identified by BLAST. Genes highlighted in bold are annotated with the 'GO' terms listed in the Results section. Genes not in bold type face were identified as having a particular function through literature searches. Genes are arranged in groups of related function. Fold changes are expressed with respect to the 'FR' animals. Gene expression is either higher (+) or lower (-) in 'FR' animals compared to 'Control' and 'Susceptible' animals (BH corrected p < 0.05).

**Table 4:** Differentially expressed (DE) genes in the expression profile of 'Fully Resistant' animals 72 hours post-inoculation

Genes grouped by function	Genbank human RefSeq ID	FR v C fold change	FR v S fold change
			
**More highly expressed in 'Fully Resistant' animals**			
			
Extracellular matrix components			
			
**Collagen, type I, alpha 1 (*COL1A1*)**	NM_000088	+2.55	+3.94
**Periostin (*POSTN*)**	NM_006475	+1.84	+3.16
			
Immunoglobulin			
			
Immunoglobulin alpha heavy chain constant region (*IGHA*)	NONE	+2.03	+1.82
			
Retinoid-binding			
			
Cellular retinoic acid binding protein 2 (*CRABP2*)	NM_001878	+1.97	+2.23
			
Other			
			
Mortality factor 4 like 1 (*MORF4L1*)	NM_206839	+1.75	+2.64
			
**Less highly expressed in 'Fully Resistant' animals**			
			
Retinoid binding			
			
**Apolipoprotein D (*APOD*)**	NM_001647	-2.04	-4.59
**Retinol binding protein 4 (*RBP4*)**	NM_006744	-2.36	-4.92
			
PPAR-γ regulators/regulated			
			
Thioredoxin interacting protein (*TXNIP*)	NM_006472	-1.87	-2.43
Nuclear protein 1 (*NUPR1*)	NM_012385	-2.06	-1.75
Pyruvate dehydrogenase kinase, isoenzyme 4 (*PDK4*)	NM_002612	-2.33	-1.89
			
Lipid mobilization and metabolism			
			
ATP-binding cassette, sub-family A, member 6 (*ABCA6*)	NM_172346	-2.69	-2.81
			
Other			
			
Ubiquitin-conjugating enzyme E2L 6 (*UBE2L6*)	NM_004223	-1.78	+2.63
Annexin A8 like 2 (*ANXA8L2*)	NM_001630	-1.84	-2.73
Pre-mRNA cleavage complex II protein (*PCF2*)	NM_015885	-1.84	-3.27
Proprotein convertase subtilisin/kexin type 6 (*PCSK6*)	NM_138319	-1.85	-2.91
Glutathione S-transferase M3 (*GSTM3*)	NM_000849	-1.91	-1.99
BTG family, member 2 (*BTG2*)	NM_006763	-1.92	-2.85
Calnexin (*CANX*)	NM_001746	-2.30	-2.03
Thymosin like 3 (*TMSL3*)	NM_183049	-5.28	-4.86
Porcine endogenous retrovirus type C (*PERV-C*)	NONE	-7.11	-3.41

A list of all DE genes identified in both the FR v C and FR v S comparisons 72 hours post-inoculation. Gene names were assigned on the basis of the best sequence match to a human gene identified by BLAST. Genes highlighted in bold are annotated with the 'GO' terms listed in the Results section. Genes not in bold type face were identified as having a particular function through literature searches. Genes are arranged in groups of related function. Fold changes are expressed with respect to the 'FR' animals. Gene expression was either higher (+) or lower (-) in 'FR' animals compared to 'Control' and 'Susceptible' animals (BH corrected p < 0.05).

**Table 5:** Selected differentially expressed (DE) genes in the expression profile of 'Susceptible' animals 24 hours post-inoculation

Genes grouped by function	Genbank human RefSeq ID	S v C fold change	S v FR fold change
			
**More highly expressed in 'Susceptible' animals**			
			
Pro inflammatory cytokine/cytokine receptors			
			
**Colony stimulating factor 3 receptor (*CSF3R*)**	NM_172313	+5.99	+5.68
**S100 calcium binding protein A9 (*S100A9*)**	NM_002965	+5.40	+6.70
**S100 calcium binding protein A12 (*S100A12*)**	NM_005621	+3.45	+3.38
Pleiotrophin (*PTN*)	NM_002825	+2.97	+2.75
**Chemokine (C-X-C motif) ligand 2 (*CXCL2*)**	NM_002089	+2.79	+2.84
			
Anti inflammatory cytokine/cytokine receptors			
			
**Interleukin 10 (*IL10*)**	NM_000572	+2.68	+4.29
**CD163 antigen (*CD163*)**	NM_203416	+2.65	+3.07
			
Neutrophil granule/phagocytosis proteins			
			
**Lactotransferrin (*LTF*)**	NM_002343	+16.47	+9.19
Matrix metalloproteinase 9 (*MMP9*)	NM_004994	+3.87	+4.89
Transcobalamin I (*TCN1*)	NM_001062	+2.55	+4.64
**Acyloxyacyl hydrolase (*AOAH*)**	NM_001637	+2.12	+2.04
			
Lipid mobilization and metabolism			
			
Resistin (*RETN*)	NM_020415	+12.77	+15.04
Solute carrier family 2 member 3 (*SLC2A3*)	NM_006931	+3.36	+2.89
Alpha-2-glycoprotein 1, zinc binding (*AZGP1*)	NM_001185	+2.83	+3.72
Lipoprotein lipase (*LPL*)	NM_000237	+2.62	+5.05
Retinol binding protein 4 (*RBP4*)	NM_006744	+2.60	+6.54
			
**Less highly expressed in Susceptible animals**			
			
Extracellular matrix components			
			
**Fibronectin 1 (*FN1*)**	NM_212482	-1.89	-2.84
**Dermatopontin (*DPT*)**	NM_001937	-2.11	-2.35
Transforming growth factor, beta-induced, (*TGFBI*)	NM_000358	-2.13	-4.68
**Microfibrillar associated protein 5 (*MFAP5*)**	NM_003480	-2.14	-2.43
**Elastin (*ELN*)**	NM_000501	-4.54	-3.82
			
Extracellular matrix growth factors			
			
Transforming growth factor beta 2 (*TGFB2*)	NM_003238	-2.35	-3.58
P311 protein (*P311*)	NM_004772	-4.14	-4.5

List of selected genes that were differentially expressed in both the S v C and S v FR comparisons 24 hours post-inoculation. Genes highlighted in bold are annotated with the 'GO' terms listed in the Results section. Genes not in bold type face were identified as having a particular function through literature searches. Genes are arranged in groups of related function. Fold changes are expressed with respect to 'Susceptible' animals. Gene expression was either higher (+) or lower (-) in 'Susceptible' animals compared to 'Control' and 'FR' animals (BH corrected p < 0.05).

**Table 6:** Selected genes that are more highly expressed in 'Susceptible' than 'Fully Resistant' and 'Control' animals at 72 hours post-inoculation

Genes grouped by function	Genbank human RefSeq ID	S v C fold change	S v FR fold change
			
Pro-inflammatory cytokine/cytokine receptors			
			
**S100 calcium binding protein A9 (*S100A9*)**	NM_002965	+8.43	+9.91
**Interleukin 1 beta (*IL1B*)**	NM_000576	+5.65	+4.01
**S100 calcium binding protein A12 (*S100A12*)**	NM_005621	+3.76	+4.4
**Chemokine (C-X-C motif) ligand 2 (*CXCL2*)**	NM_002089	+2.45	+2.32
**Interleukin 8 (*IL8*)**	NM_000584	+2.21	+3.45
			
Anti-inflammatory cytokine/cytokine receptors			
			
**CD163 antigen (*CD163*)**	NM_203416	+2.71	+2.49
**Interleukin 1 receptor antagonist (*IL1RN*)**	NM_173843	+2.31	+2.70
Interleukin 4 receptor (*IL4R*)	NM_000418	+2.27	+1.93
			
Neutrophil granule/phagocytosis proteins			
			
**Cathelicidin antimicrobial peptide (*CAMP*)**	NM_004345	+5.30	+10.41
Matrix metalloproteinase 9 (*MMP9*)	NM_004994	+5.09	+4.19
Transcobalamin I (*TCN1*)	NM_001062	+4.48	+4.10
Chitinase 3-like 1 (*CHI3L1*)	NM_001276	+4.48	+4.84
**Solute carrier family 11 member A1 (*SLC11A1*)**	NM_000578	+3.38	+2.54
Cathepsin L (*CTSL*)	NM_145918	+3.19	+3.01
**Neutrophil cytosolic factor 1 (*NCF1*)**	NM_000265	+2.61	+2.07
**Cytochrome b-245, beta polypeptide (*CYBB*)**	NM_000397	+2.11	+1.96
**Ras-related C3 botulinum toxin substrate 1 (*RAC1*)**	NM_006908	+2.07	+1.86
Cathepsin C (*CTSC*)	NM_148170	+2.07	+2.29
			
Lipid mobilization and metabolism			
			
Resistin (*RETN*)	NM_020415	+12.96	+12.24
Adrenergic, alpha-2A-, receptor (*ADRA2A*)	NM_000681	+3.71	+3.85
**Lipoprotein lipase (*LPL*)**	NM_000237	+3.70	+4.54
Benzodiazapine receptor (peripheral) (*BZRP*)	NM_000714	+2.55	+3.10
Apolipoprotein D (*APOD*)	NM_001647	+2.25	+4.59
PDZK1 interacting protein 1 (*PDZK1IP1*)	NM_005764	+2.15	+2.48
Angiopoietin-like 4 (*ANGPTL4*)	NM_139314	+2.14	+3.07
Retinol binding protein 4 (*RBP4*)	NM_006744	+2.10	+4.94
			
Lipid biosynthesis			
			
Solute carrier family 2, member 3 (*SLC2A3*)	NM_006931	+4.03	+4.11
**Leukotriene A4 hydrolase (*LTA4H*)**	NM_000895	+3.11	+3.28
**Thromboxane A synthase 1 (*TBXAS1*)**	NM_030984	+2.24	+1.97
**Prostaglandin-endoperoxide synthase 1 (*PTGS1*)**	NM_080591	+1.95	+2.11
**Arachidonate 5-lipoxygenase-activating protein (*ALOX5AP*)**	NM_001629	+1.94	+2.35
**Acyl-CoA synthetase long-chain family member 4 (*ACSL4*)**	NM_022977	+2.07	+2.06
**1-acylglycerol-3-phosphate O-acyltransferase 6 (*AGPAT6*)**	NM_178819	+2.31	+2.52
			
Coagulation			
			
**Thrombospondin 1 (*THBS1*)**	NM_003246	+2.79	+3.36
**Serine proteinase inhibitor, clade E, member 1 (*SERPINE1*)**	NM_000602	+2.49	+2.55
Adaptor-related protein complex 3, delta 1 subunit (*AP3D1*)	NM_003938	+2.24	+2.28
Tumor necrosis factor, alpha-induced protein 6 (*TNFAIP6*)	NM_007115	+2.63	+2.90
**Thromboxane A synthase 1 (*TBXAS1*)**	NM_030984	+2.24	+1.97
**Phospholipid scramblase 4 (*PLSCR4*)**	NM_020353	+2.02	+2.86

List of selected genes that are more highly expressed in 'Susceptible' than 'FR' and 'Control' animals 72 hours post-inoculation. Genes are arranged in groups of related function. Genes highlighted in bold are annotated with the 'GO' terms listed in the Results section. Genes not in bold type face were identified by as having a particular function through literature searches. Fold changes are expressed with respect to 'Susceptible' animals (BH corrected p < 0.05).

**Table 7:** Selected genes that are less highly expressed in 'Susceptible' than 'Fully Resistant' and 'Control' animals at 72 hours post-inoculation

Genes grouped by function	Genbank human RefSeq ID	S v C fold change	S v FR fold change
			
Extracellular matrix components			
			
Fibronectin type III domain containing 1 (*FNDC1*)	NM_032532	-1.78	-1.86
**Collagen, type VI, alpha 3 (*COL6A3*)**	NM_057167	-1.82	-2.52
Fibronectin type III domain containing 3 (*FNDC3*)	NM_014923	-1.83	-2.46
**Decorin (*DCN*)**	NM_133503	-1.98	-1.88
**Extracellular matrix protein 2 (*EMP2*)**	NM_001393	-2.18	-2.50
EGF-containing fibulin-like extracellular matrix protein 1 (*EFEMP1*)	NM_018894	-2.2	-2.78
**Secreted protein, acidic, cysteine-rich (*SPARC*)**	NM_003118	-2.2	-2.78
**Matrilin 2 (*MATN2*)**	NM_030583	-2.35	-2.7
**Collagen, type I, alpha 1 (*COL1A1*)**	NM_000088	-2.38	-3.39
**Collagen, type III, alpha 1 (*COL3A1*)**	NM_000090	-2.52	-3.65
**Fibronectin 1 (*FN1*)**	NM_212482	-2.52	-3.29
**Dermatopontin (*DPT*)**	NM_001937	-2.73	-2.75
**Transforming growth factor, beta-induced (*TGFBI*)**	NM_000358	-3.02	-3.79
**Lectin, galactoside-binding, soluble, 3 binding protein (*LGALS3BP*)**	NM_005567	-3.2	-3.09
Asporin (*ASPN*)	NM_017680	-3.38	-3.66
**Microfibrillar associated protein 5 (*MFAP5*)**	NM_003480	-5.54	-4.58
			
ECM growth factor ligand/receptor			
			
Bone morphogenetic protein receptor, type II (*BMPR2*)	NM_033346	-1.76	-2.07
Insulin-like growth factor binding protein 5 (*IGFBP5*)	NM_000599	-1.76	-2.17
Fibroblast growth factor 17 (*FGF17*)	NM_003867	-2.03	-2.72
**WNT family, member 5A (*WNT5A*), mRNA**	NM_003392	-2.16	-2.14
P311 protein (*P311*)	NM_004772	-6.98	-9.01
			
Antigen presentation			
			
**Proteasome subunit, beta type, 8 (*PSMB8*)**	NM_148919	-2.06	-1.83
**CD1B antigen, b polypeptide (*CD1B*)**	NM_001764	-2.11	-2.40
**Major histocompatibility complex, class II, DM alpha (*HLA-DMA*)**	NM_006120	-2.18	-1.83
**Major histocompatibility complex, class II, DR alpha (*HLA-DRA*)**	NM_019111	-2.3	-2.31
**Cathepsin S (CTSS)**	NM_004079	-2.32	-1.94
**Major histocompatibility complex, class II, DM beta (*HLA-DMB*)**	NM_002118	-3.21	-2.93
			
T cell receptor signaling			
			
T cell receptor alpha locus (*TCRA*)	NONE	-2.45	-3.34
Dual specificity phosphatase 14 (*DUSP14*)	NM_007026	-2.50	-1.94
V-set and immunoglobulin domain containing 4 (*VSIG4*)	NM_007268	-2.91	-2.00
GATA binding protein 3 (*GATA3*)	NM_002051	-3.00	-3.22
			
Immunoglobulin genes			
			
**Immunoglobulin J polypeptide (*IGJ*)**	NM_144646	-1.90	-2.73
Immunoglobulin kappa chain variable region (*IGK*)	NONE	-2.36	-5.05
			
Anti-viral and type I interferon regulated genes			
			
**Interferon regulatory factor 9 (*IRF9*)**	NM_006084	-1.82	-2.01
Hect domain and RLD 5 (*HERC5*)	NM_016323	-1.85	-1.95
Interferon regulatory factor 2 binding protein 2 (*IRF2BP2*)	NM_182972	-1.90	-1.95
**Interferon induced transmembrane protein 3 (*IFITM3*)**	NM_021034	-2.52	-2.08
**Tripartite motif protein TRIM5 isoform alpha (*TRIM5A*)**	NM_033034	-3.02	-3.00
**Myxovirus resistance 1 (*MX1*)**	NM_002462	-3.26	-2.58
Interferon-induced protein 44 (*IFI44*)	NM_006820	-3.26	-2.98
Family with sequence similarity 14, member A (*FAM14A*)	NM_032036	-3.75	-2.89
Ubiquitin specific protease 18 (*USP18*)	NM_017414	-3.84	-2.65
**2',5'-oligoadenylate synthetase 1 (*OAS1*)**	NM_002534	-3.87	-3.21
Interferon-induced protein with tetratricopeptide repeats 1 (*IFIT1*)	NM_001001887	-4.99	-3.04
**DEAD box polypeptide 58 (*DDX58*)**	NM_014314	-5.30	-3.70
XIAP associated factor-1 (*XIAPAF1*)	NM_017523	-6.07	-5.08
**ISG15 ubiquitin like modifier (*ISG15*)**	NM_005101	-6.82	-3.85
**Interferon, alpha-inducible protein 6 (*IFI6*)**	NM_002038	-7.18	-3.63
**2'-5'-oligoadenylate synthetase 2 (*OAS2*)**	NM_016817	-9.55	-8.96

List of selected genes that are less highly expressed in 'Susceptible' than 'FR' and 'Control' animals 72 hours post-inoculation. Genes are arranged in groups of related function. Genes highlighted in bold are annotated with the 'GO' terms listed in the Results section. Genes not in bold type face were identified by as having a particular function through literature searches. Fold changes are expressed with respect to 'Susceptible' animals (BH corrected p < 0.05).

### Expression profile of 'FR' animals

In total, 28 transcripts representing 21 genes were found to be differentially expressed between 'FR' animals and 'Control' and 'Susceptible' animals at 24 hours. Expression of 13 genes was higher in 'FR' animals than 'Susceptible' and 'Control' animals (FR/C and FR/S), while expression of 8 genes was lower. Human GO annotation was available for 18 genes. At 72 hours, 21 transcripts representing 20 genes were differentially expressed, of which 17 had associated GO annotation. Expression of 5 of these genes was higher in FR/C and FR/S whereas expression of 15 genes was lower. Lists of the DE genes that constitute the 'FR' expression profile at the 24 and 72 hour time-points are presented in Tables 3 and 4 respectively. Two genes, *APOD *and *RBP4*, are common to both lists.

Eight of the genes whose expression was higher in FR/C and FR/S at 24 hours are associated with the GO annotation term 'proteinaceous extracellular matrix' (GO:0005578). This GO term was found to be statistically over-represented among genes whose expression was higher in 'FR' animals at 24 hours (p = 1.33 × 10^-11^). The genes are *COL1A2*, *COL3A1*, *COL6A3*, *COL15A1, EFEMP2*, *LTBP4*, *TGFBI*, and *TNC*. Four of these genes encode types of collagen. Two genes whose expression was lower in FR/C and FR/S at 24 hours, *APOD *and *RBP4*, encode lipocalin molecules. These two genes are associated with the GO molecular function ontology term 'retinol binding' (GO:0005501) that was significantly over-represented among genes whose expression was lower in 'FR' animals (p = 3.16 × 10^-4^). Another term that was significantly enriched among this group of genes was 'response to unfolded protein' (GO:0006986; p = 1.98 × 10^-4^). Two heat shock proteins in the list, *DNAJB1 *and *HSPA6*, are associated with this term.

At 72 hours, two genes whose expression was higher in FR/C and FR/S were proteinaceous extracellular matrix (ECM) components: *COL1A1 *and *POSTN*. As at 24 hours, this term was found to be statistically over-represented among the group of genes whose expression was higher in 'FR' animals at this time-point (p = 9.13 × 10^-3^). Another more highly expressed transcript matched the coding sequence of the constant region of immunoglobulin A (IgA). Expression of the lipocalin genes *APOD *and *RBP4 *was lower in FR/C and FR/S at 72 hours, as it was at 24 hours. Again, the term 'retinol binding' was significantly enriched in the gene list (p = 2.25 × 10^-3^). Literature searches revealed that several of the genes whose expression was lower in FR/C and FR/S have been shown to either regulate or be regulated by the lipid-binding transcription factor Peroxisome proliferator-activated receptor gamma (PPAR-γ) in man. These genes are *NUPR1*, *PDK4*, and *TXNIP *[22-24]. Other DE genes which function in retinoid and lipid transport are *ABCA6 *and *CRABP2*.

### Expression profile of 'Susceptible' animals

In the 'Susceptible' animals at 24 hours, a total of 60 transcripts representing 58 genes were found to be differentially expressed compared to both 'Control' and 'Fully Resistant' pigs. Human GO annotation was available for 53 genes. Expression of 32 genes was higher in S/C and S/FR whereas expression of 21 genes was lower. Approximately 60% of these genes were also found to be differentially expressed at 72 hours in the same direction, indicating that there is considerable similarity in expression profiles at the two time-points. At the 72 hour time-point, 384 transcript sequences from 347 genes were differentially expressed, with human GO annotation available for 293 genes. Expression of 136 genes was higher in S/C and S/FR whereas expression of 248 genes was lower. Lists of selected DE genes in the expression profile of 'Susceptible' animals at 24 and 72 hours post-inoculation are provided in Tables 5, 6, and 7.

At 24 hours, several GO terms associated with the immune system were significantly over-represented among the group of genes whose expression was higher in 'Susceptible' animals than both 'Control' and 'FR' animals (S/C and S/FR). These were 'defence response' (GO:0006592; p = 9.3 × 10^-6^), 'inflammatory response' (GO:0006954; p = 2.88 × 10^-5^), 'response to wounding' (GO:0009611; p = 2.04 × 10^-4^), 'response to bacterium' (GO:0009617; p = 6.30 × 10^-3^), and 'leukocyte chemotaxis' (GO:0030595; p = 8.28 × 10^-3^). A large proportion of the most highly differentially expressed genes are associated with an acute inflammatory response to bacterial infection. Three groups of genes of related immune function were identified through literature searches as having higher expression in S/C and S/FR. The first group was cytokines and their receptors. Most of these either promote the functional activities of neutrophils, including the phagocytosis of bacteria (such as *CSF3R*), or are neutrophil chemokines (such as *S100A9*) [25,26]. However, two anti-inflammatory cytokine genes were also identified: *CD163 *and *IL10*. A second group of genes encode neutrophil granule proteins, such as the antimicrobial proteins *AOAH *and *LTF*, and the secreted protease *MMP9 *[27]. The third group contains genes involved in the uptake and metabolism of lipids. It includes the most differentially expressed gene in 'Susceptible' animals, *RETN*, as well as *LPL *and *RBP4*, two genes whose expression was lower in FR/C and FR/S.

The principal over-represented GO term among genes whose expression was lower in S/C and S/FR was 'proteinaceous extracellular matrix' (GO:0005578; p = 1.04 × 10^-3^). Interestingly, as stated previously, this term was associated with genes whose expression was higher in FR/C and FR/S at 24 and 72 hours. The 5 genes annotated under this term were *DPT*, *ELN*, *FN1*, *MFAP5*, and *TGFBI*, and additional ECM components were also identified in the list by literature searches. Expression of *TGFB2*, a member of the transforming growth factor beta family of genes that promote tissue repair, was also lower in S/C and S/FR.

Many of the most statistically significant GO terms at 24 hours were also over-represented at 72 hours, but a larger number of genes were annotated under each GO term for the latter, a reflection of the increased number of DE genes identified at this time-point. GO terms associated with immunology were 'defence response' (GO:0006592; p = 5.60 × 10^-10^), 'inflammatory response' (GO:0006954; p = 4.94 × 10^-11^), 'response to bacterium' (GO:0009617; p = 1.42 × 10^-3^), 'acute phase response' (GO:0006953; p = 4.88 × 10^-3^), 'leukocyte chemotaxis' (GO:0030595; p = 2.46 × 10^-3^), and 'response to molecule of bacterial origin' (GO:0002237; p = 9.42 × 10^-3^). Among the list of genes whose expression was higher in S/C and S/FR at 72 hours were additional cytokine genes, including the well-characterized pro-inflammatory genes *IL1B *and *IL8*, and the anti-inflammatory *IL1RN*, whose protein is an antagonist of IL-1β [28]. Several other genes encoding neutrophil granule proteins were identified, such as *CAMP *and *CHI3L*, as were 3 components of the neutrophil NADPH oxidase that participates in phagocytosis (*CYBB*, *NCF1*, and *RAC1*) [29]. More genes involved in lipid metabolism were also found to be differentially expressed in 'Susceptible' animals at this time-point. The GO terms 'fatty acid metabolic process' (GO:0006631; p = 3.12 × 10^-4^), 'icosanoid biosynthetic process' (GO:0046456; p = 9.42 × 10^-3^), and 'prostaglandin biosynthetic process' (GO:0001516; p = 9.42 × 10^-3^) were all over-represented in the expression profile of 'Susceptible' animals. The list includes genes for enzymes that function in the biosynthesis of the potent lipid inflammatory mediators leukotriene B_4 _(the genes *LTA4 H *and *ALOX5AP*) and thromboxane A2 (the genes *TBXAS1 *and *PTGS1*). Finally, the GO term 'coagulation' (GO:0050817; p = 8.78 × 10^-3^) was significantly over-represented. Genes annotated with this term include the protease inhibitor *SERPINE1 *and the adhesive glycoprotein *THSB1 *[30,31].

Two groups of related GO terms were statistically over-represented in the list of genes whose expression was lower in S/C and S/FR. The first group included terms relating to ECM, which were also identified in the expression profile of 'Susceptible' animals at 24 hours. These were 'proteinaceous extracellular matrix' (GO:0005578; p = 5.83 × 10^-6^) and 'extracellular matrix part' (GO:0044420; p = 3.24 × 10^-3^). The second group were immunology terms: 'immune response' (GO:0006955; p = 1.15 × 10^-3^), 'defence response' (GO:0006952; p = 7.18 × 10^-3^), and 'response to virus' (GO:0009615; p = 8.51 × 10^-4^). Although the term 'defence response' was also over-represented among genes whose expression was higher in S/C and S/FR, the genes annotated with this all encompassing term had different functions. Broadly speaking, two different groups of genes whose expression was lower in S/C and S/FR were identified: those involved in antigen presentation to T cells and those that functioned in antiviral immunity. The first group contains genes whose products function in either class I or class II presentation of peptide antigens or presentation of lipid antigens. Those genes implicated in the former are *PSMB8 *(class I), and *CTSS*, *HLA-DMA*, *HLA-DMB*, and *HLA-DRA *(class II) [32,33]. *CD1B *presents lipid antigens. Two transcripts encoding putative immunoglobulin J and kappa chains were also found to be less highly expressed in the 'Susceptible' group. The second, and largest, group contains genes whose products have known antiviral activity such as *MX1 *and *OAS2 *[34,35]. It also includes genes that are transcribed in response to signalling by the antiviral type I interferons (e.g. *HERC5 *and *IFITM3*) [36,37].

### Verification of microarray data by Reverse Transcription Quantitative Polymerase Chain Reaction (RT-qPCR)

**Figure 2:**
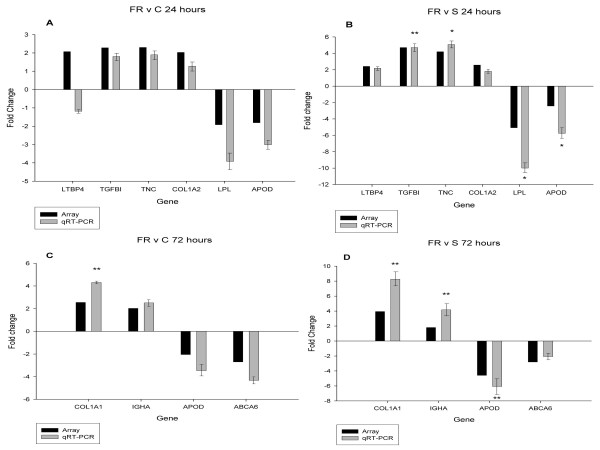
**Comparison of relative expression differences for selected genes from the 'Fully Resistant' expression profiles determined by microarray and RT-qPCR**. The relative expression fold change for genes identified as being differentially expressed in the (A) FR v C comparison at 24 hours, (B) FR v S comparison at 24 hours, (C) FR v C comparison at 72 hours, and (D) FR v S comparison at 72 hours. For each gene, the black bar indicates the fold change as determined by microarray; the grey bar indicates the expression ratio as determined by RT-qPCR, with associated standard error bars. Relative expression ratios are given as the ratio of the first group compared to the second (e.g. 'Fully Resistant' compared to 'Control' in the graph in panel A). Statistical significance of the relative expression ratio is indicated (* p < 0.05; ** p < 0.01). Results for *TMSL3 *at 72 hours were not plotted on graphs C and D because of the axis scale chosen. The array fold changes for this gene in the FR v C and FR v S comparisons were -5.26 and -4.85 whereas the RT-qPCR fold changes were -101.75 and -73.47 respectively, the last being statistically significant (p < 0.05). Primer information is provided in additional file 5.

The microarray results were verified for selected DE genes by RT-qPCR. A list of the genes and their associated primer sequences is provided in additional file 5. RT-qPCR was carried out for 23 genes selected from the expression profiles of 'FR' and Susceptible animals. These genes had been identified as being differentially expressed in at least two of the comparisons by microarray analysis (FR v C and FR v S, or S v C and S v FR at 24 or 72 hours post-inoculation). The microarray and RT-qPCR fold changes for DE genes in the 'FR' profile at 24 and 72 hours are shown in Figure 2. Four out of 6 genes tested at 24 hours, produced a normalised expression ratio of > 1.75:1, in agreement with the microarray data for both comparisons (FR v C, and FR v S). None of these differences reached statistical significance for the FR v C comparison, whereas 4 out of 6 did in the FR v S comparison. At 72 hours, all 10 RT-qPCR results were in agreement with the microarray data on fold-change, with 5 out of 10 being statistically significant.

**Figure 3:**
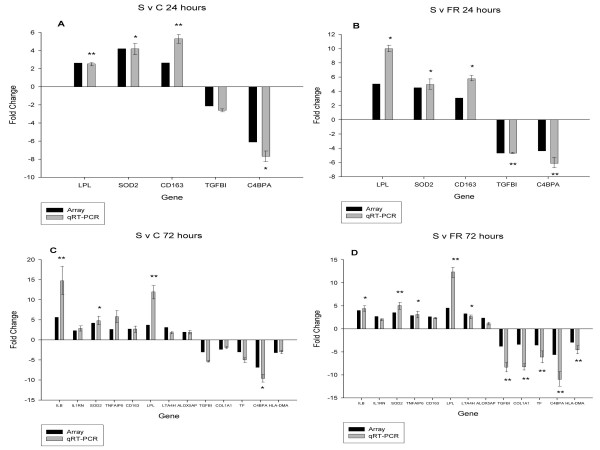
**Comparison of relative expression differences for selected genes from the 'Susceptible' expression profiles determined by microarray and RT-qPCR**. The relative expression fold change for genes identified as being differentially expressed in the (A) S v C comparison at 24 hours, (B) S v FR comparison at 24 hours, (C) S v C comparison at 72 hours, and (D) S v FR comparison at 72 hours. For each gene, the black bar indicates the expression ratio as determined by microarray; the grey bar indicates the expression ratio as determined by RT-qPCR, with associated standard error bars. Relative expression ratios are given as the ratio of the first group compared to the second (e.g. 'Susceptible' compared to 'Control' in the graph in panel A. Statistical significance of the relative expression ratio is indicated (* p < 0.05; ** p < 0.01). Results for *RETN *and *LTF *at 24 hours, and *RETN *and *OAS2 *at 72 hours were not plotted because of the axis scale chosen. The array fold changes for *RETN *and *LTF *at 24 hours in the S v C comparison were +12.77 and +16.45 whereas the RT-qPCR fold changes were +53.65 and +24.69 respectively. For the S v FR comparison at 24 hours the array fold changes were +14.93 and +9.13 whereas the RT-qPCR changes were +38.38 and +16.68 respectively. For the S v C comparison at 72 hours, the array fold changes for *RETN *and *OAS2 *were +13.00 and -9.58 whereas the RT-qPCR fold changes were +98.77 and -26.31 respectively. For the S v FR comparison at 72 hours, the array fold changes were +12.24 and -3.58 whereas the RT-qPCR fold changes were +66.20 and -20.00 respectively. All fold changes for *RETN*, *LTF*, and *OAS2 *measured by RT-qPCR were statistically significant (p < 0.01). Primer information is provided in additional file 5.

RT-qPCR results for DE genes in the 'Susceptible' profile are shown in Figure 3. All 7 of the genes tested at 24 hours were differentially expressed with a ratio of > 1.75:1; this ratio was significant in 13 out of 14 cases. At 72 hours, the direction of differential expression was in agreement for all 15 genes across both the S v C and S v R comparisons, with 18 out of 30 being statistically significant. Linear regression analysis revealed a strong correlation between the microarray and RT-qPCR data for each of the comparisons, with r^2 ^coefficients ranging from 0.83 to 0.99 (data not shown).

## Discussion

For this study, microarray experiments were carried out to compare gene expression in lung tissue of animals that were 'Fully Resistant' or 'Susceptible' to Glässer's disease, together with mock-inoculated 'Control' animals, at 24 and 72 hours post-inoculation. This was done with the principal aim of identifying candidate genes responsible for the observed differences in susceptibility, but also to increase knowledge of the genetic programmes activated in the host during *H. parasuis *infection in affected animals. 'Fully Resistant' and 'Susceptible' animals were matched for sire and time-point to reduce background differences in gene expression not attributable to differences in infection status. Unfortunately, it was not possible to match multiple mock-inoculated 'Control' animals to 'FR' and 'Susceptible' animals for both sire and time-point, as only one animal from each sire was designated as a 'Control' animal at each time-point in the original challenge experiment. Instead, pools were created from lung RNA from 'Control' animals of the same sire but different time-points (24, 48, and 72 hours). It is possible therefore that some of the DE genes identified in the 'FR v C' and 'S v C' comparisons could be attributable to a 'day effect' that is independent of differences caused by exposure to *H. parasuis*. However, this is not the case for the 'FR v S/S v FR' comparison, and as the expression profiles described in the Results section only contain genes that were differentially expressed between the group of interest and both other groups (i.e. for the 'FR' expression profile, genes identified as being differentially expressed in FR v C and FR v S comparisons; for the 'Susceptible' expression profile, genes identified as being DE in the S v C and S v FR comparisons), genes whose differential expression is attributable to such a 'day effect' should be absent from the expression profiles.

Overall, the microarray experiments successfully identified differentially expressed (DE) genes in comparisons between 'FR', 'Susceptible', and 'Control' animals, as determined by the good concordance observed between fold changes determined by microarray and RT-qPCR. Fold changes for some of the genes did not reach statistical significance when measured by RT-qPCR, particularly for the 24 hour time-point where only 2 biological replicates were available. However, the direction of expression differences was the same for 21 out of the 23 genes tested.

The results showed that fewer genes were differentially expressed between 'FR' and 'Control' animals (FR v C) than between 'Susceptible' animals and these two groups (S v C and S v FR). This finding was expected as the basis for the 'FR' susceptibility classification was the absence of any signs of an ongoing *H. parasuis *infection in inoculated pigs, and none of the mock-inoculated 'Control' pigs were infected. RT-PCR confirmed the absence of detectable amounts of *H. parasuis *in both 'FR' and 'Control' animals. In contrast, all 'Susceptible' animals exhibited multiple signs of infection and had detectable levels of *H. parasuis *in lung tissue.

To aid the identification of genes and pathways that could be involved in disease susceptibility phenotypes, expression profiles of the 'FR' and 'Susceptible' animals were generated for both time-points. These profiles contained only those genes that were differentially expressed between the group of interest and both other groups, and therefore potentially contained genes that were regulated differently in 'FR' and 'Susceptible' animals after exposure to *H. parasuis*. The expression profile of 'FR' animals at 24 hours provided some evidence that this group of animals had rapidly cleared bacteria from the lungs and progressed to the repair of tissue damaged during the localized response to infection. Eight of the 13 genes whose expression was higher in FR/C and FR/S at 24 hours encode ECM proteins. Moreover, a number of these genes have previously been shown to be transcribed in response to signalling by members of the TGF-β family, the main group of cytokines that co-ordinate the repair response [38-42].

Expression of fewer ECM components was higher in FR/C and FR/S at 72 hours which might indicate that the repair process had largely finished by this time. One transcript whose expression was higher in 'FR' animals was identical in sequence to the heavy chain constant region of swine IgA, and the differential expression of this gene was confirmed by RT-qPCR (see Figure 2). The important role that IgA plays in protecting the host against infection at mucosal surfaces is well documented [43]. The short time-course of this experiment makes it unlikely that this antibody could have been generated through an adaptive immune response to infection. One possibility is that this gene encodes a 'natural antibody'. Natural antibodies are high-avidity, low-affinity molecules that bind conserved pathogen associated molecular patterns (PAMPs) [44]. Their production does not require T-cell help, so they are produced more quickly in response to microbes than classical antibodies, and are considered to be components of the innate immune system. Although natural antibodies are principally of the IgM class, natural IgA has been found at mucosal surfaces in other species [45,46]. Interestingly, the polymeric immunoglobulin receptor (*PIGR*) gene, required for the transport of secretory IgA to mucosal surfaces, was found to be upregulated in 'FR' compared to 'Control' animals at 24 hours (see additional file 1). It is likely that natural antibodies are important in protecting the neonatal piglet from infection during a time at which their adaptive immune systems are incompletely developed [47]. However, the existence of a natural antibody producing subset of cells, such as the B1 cell characterized in mice, has yet to be formally demonstrated in swine [48]. The titres of neutralizing antibodies found in neonatal animals have a genetic component, as demonstrated by variation in the levels of natural antibody to vesicular stomatitis virus found in the sera of different inbred mouse strains [49]. Consequently, polymorphisms in genes that control antibody production could constitute one mechanism influencing susceptibility to *H. parasuis *infection.

A group of genes whose expression was lower in FR/C and FR/S at 72 hours are involved in the transport and metabolism of retinoids and lipids. This includes a group of genes whose proteins either regulate, or are regulated by, the transcription factor PPAR-γ. PPAR-γ, a member of the PPAR family of lipid-binding nuclear hormone receptors, functions as a heterodimer with retinoid X receptors (RXRs) [50]. This transcription factor was first identified in man for its role in the differentiation of adipocytes, but has subsequently also been shown to be a negative regulator of pro-inflammatory (TNF-α, IL-1β) signalling by macrophages [51,52]. In macrophages, activation of PPAR-γ promotes the uptake of lipids in the short term, in part through upregulation of the scavenger receptor gene *CD36*. However, the actions of CD36 in lipid uptake appear to be balanced by lipid efflux co-ordinated by the PPAR-γ target gene Liver X Receptor (*LXR*) and effected by ABC type transporters [53]. The mechanism by which PPAR-γ blocks pro-inflammatory signalling is not known, but the absence of response elements in genes repressed by PPAR-γ agonists indicates that it is probably by trans-repression of selected NF-κB and AP-1 responsive genes [54].

The gene expression profile of the 'FR' animals at 72 hours post-inoculation points to a reduction in PPAR-γ signalling, which would be expected to promote pro-inflammatory activation of macrophages. One possibility is that the expression profile reflected a basal difference in macrophage gene expression which predisposed these animals to a more rapid and effective antimicrobial response at the critical early stage of infection. If this were the case then genes involved in PPAR-γ signalling would be promising candidates for disease resistance phenotypes. An alternative explanation is that PPAR-γ signalling was actively being switched off as the tissue repair mechanisms evident in the 'FR' animals at 24 hours were completed. PPAR-γ controls the 'alternative' activation of macrophages characterized by the release of anti-inflammatory cytokines such as TGF-β during tissue repair [55]. Irrespective of whether a reduction in PPAR-γ signalling is a cause or effect of the 'Fully Resistant' phenotype, several of the most highly expressed genes in 'FR' animals compared to 'Control' animals encode components of the innate immune system. These include the scavenger receptor 'Deleted in malignant brain tumour 1' (*DMBT1*) and peptidoglycan recognition protein 1 (*PGLYRP1*) (see additional file 1) [56,57]. In addition, the role that natural antibodies may play has already been discussed. Overall, these data support the hypothesis that the 'Fully Resistant' animals actively cleared *H. parasuis *from the lungs before it could be disseminated to other sites in the body. An inevitable issue with this experiment was that in order to confidently categorize *H. parasuis*-inoculated animals as 'Fully Resistant', the animals had to be demonstrably disease-free and have no detectable bacteria remaining in the lungs. However, this approach precludes the investigation of gene expression at earlier time-points when bacteria are still present, which is unfortunate as this is likely to be a time of critical importance in determining the ultimate outcome of infection. Determining the genomic response to *H. parasuis *during these very early stages of infection would help to identify more gene candidates for resistance to Glässer's disease, albeit with the drawback that it would not be possible to classify the animals used into susceptibility categories.

In contrast to the 'FR' expression profile, many DE genes were identified in the expression profile of 'Susceptible' animals, especially at the 72 hour time-point. The profiles contain many genes whose differential expression is likely to be a consequence of an ongoing host response to *H. parasuis *infection. The most striking feature was the marked increase in the expression of genes associated with inflammation: either encoding components of signalling pathways associated with it (e.g. cytokines, cytokine receptors), or proteins that function in the various biological processes encompassed by the term (e.g. neutrophil phagocytosis, coagulation). This finding is in agreement with the fibrino-purulent pleuritis observed in the lungs of 'Susceptible' animals [14]. The biological effects of many of the differentially expressed signalling molecules are mediated by regulation of the transcription factors NF-κB and AP-1. Interestingly, both pro and anti-inflammatory cytokine, cytokine receptors, and intracellular components of these signalling pathways are on the lists of differentially expressed genes in the 'Susceptible' expression profiles. To use signalling through the IL-1 receptor as an example; the gene for the pro-inflammatory cytokine IL-1β was more highly expressed in 'Susceptible' animals at 72 hours. However, the gene encoding its antagonist, IL-1RN, was also more highly expressed, as was the gene *NFKBIA*, whose product inhibits the NF-κB complex. In addition, the gene for Caspase-1 (*CASP1*), which is required to process the IL-1β pro-protein into its active form, was much less highly expressed in 'Susceptible' animals. In rats, expression of both *IL-1B *and *IL1-RN *is induced by lipopolysaccharide (LPS), and the kinetics of their activation indicates that IL-1RN likely acts as an endogenous negative feedback regulator of IL-1β [58]. An imbalance of IL-1β and IL1-RN signalling has been proposed as an important factor in several inflammatory conditions in man such as asthma and arthritis [59,60]. It is noticeable that the most highly expressed genes in 'Susceptible' compared to 'Control' and 'FR' animals (such as *IL1B*, *RETN*, and *S100A9*) encode cytokines that promote inflammation. This suggests that endogenous regulation mechanisms failed to keep the inflammatory response in check in these animals, likely due to pro-inflammatory stimuli caused by the continued presence of *H. parasuis*.

An increase in pro-inflammatory cytokine gene expression was also observed in the lungs of pigs infected by *Actinobacillus pleuropneumoniae*, another respiratory pathogen of pigs that is related to *H. parasuis *[15], and the spleen of pigs infected with *H. parasuis *[17]. The specific pro-inflammatory cytokines identified differ considerably between the three studies, likely due to the differences in the microarrays used and host-pathogen interactions. Common genes between studies were the neutrophil chemokine *IL8*, identified in both this study and that of Hedegaard *et al. *[15], and the S100 proteins S100A9 and S100A12, and the resistin (*RETN*) gene, which were all highly upregulated in response to *H. parasuis *in the lung and spleen. Indeed, *RETN *was the most upregulated gene in both our study and that of Chen *et al. *[17]. First identified for its role in adipocyte differentiation, its expression in leukocytes and function as a pro-inflammatory cytokine has only recently been recognized [61,62]. This study identified a number of other genes in addition to *RETN *that function in the mobilization and biosynthesis of lipids, and which are more highly expressed in 'Susceptible' animals. Several of these genes have also recently been shown to have roles in human inflammatory disorders. For example, *LPL *and *SLC2A3 *are expressed in the "foamy" macrophages of atherosclerotic plaques [63,64]. These results implicate *RETN *and other lipid metabolism genes in the pathogenesis of Glässer's disease. Novel drugs targeting these molecules could be promising treatments for reducing the severe immunopathology associated with this, and perhaps other, infectious diseases in the future.

Some genes whose expression is higher in S/C and S/FR encode proteins of known antimicrobial activity. Mutations affecting the function of any of these genes could increase susceptibility to Glässer's disease. Lactotransferrin (*LTF*) has both bacteriostatic and bactericidal properties [65,66]. Although activity against *H. parasuis *has not been demonstrated, *LTF *has been shown to attenuate the pathologic potential of a related bacterium, *Haemophilus influenzae*, a respiratory pathogen of man [67]. Other genes with products of known antimicrobial activity are *CAMP*, *PTX3*, and *SLC11A1 *[68-70].

Genes whose expression was lower in S/C and S/FR include those that function in class I or class II antigen presentation, T cell function, and immunoglobulin production. Again, our results corroborate those obtained by Hedegaard *et al. *[15] and Chen *et al. *[17], who also found a reduction in expression of genes whose products function in antigen presentation, albeit the identities of the genes are different. During *A. pleuropneumoniae *infection, the MHC genes *HLA-DRA *and *HLA-DQA1 *were found to be downregulated whereas in the spleen of animals infected with *H. parasuis *it was *HLA-B *and *HLA-DRB1*. In this study the differentially expressed MHC genes identified were *HLA-DMA*, *HLA-DMB*, and *HLA-DRA*. A reduction in the capability of pigs to mount both cell-mediated and antibody based responses to *H. parasuis *would clearly be expected to have a detrimental effect on the ability of these animals to clear infection. Chen *et al. *postulated that these changes could be a consequence of an immune evasion strategy employed by *H. parasuis*, and this is certainly a possibility that warrants further research. An alternative explanation supported by our results is that the observed immunosuppression in the 'Susceptible' group is caused by an increased production of IL-10, as this cytokine strongly inhibits both class I and class II antigen presentation [71,72]. *IL10 *gene expression was increased in 'Susceptible' compared to 'Control' animals at both 24 and 72 hours (see additional files 1 and 2). One possibility is that this is a consequence of 'endotoxin tolerance', in which cells, particularly macrophages, become desensitised to repeated stimulation by LPS. Tolerized cells upregulate the expression of anti-inflammatory genes such as *IL-10*, *IL1-RN*, or *TGFB1 *[73]. Endotoxin tolerance is an important mechanism for regulating an excessive inflammatory response, but it comes at the cost of preventing the induction of strong adaptive immune responses to infection. An increase in the amount of IL-10 in the lung could also account for the decrease in expression of genes regulated by type I interferons. IL-10 inhibits production of IFN-α in peripheral blood mononuclear cells in man in response to herpes simplex virus (HSV) and vesicular stomatis virus (VSV) [74,75].

## Conclusions

This study has identified gene expression profiles associated with host resistance and susceptibility to infection by *H. parasuis *in CD piglets. The results support a scenario in which 'Fully Resistant' animals rapidly eliminated *H. parasuis *from the lung through an effective innate immune response. The failure of 'Susceptible' animals to resolve the infection at an early stage resulted in the systemic spread of the bacterium and the development of lesions and clinical signs associated with Glässer's disease. Patterns of gene expression associated with features of the molecular pathology of the disease were identified in 'Susceptible' pigs: namely neutrophil infiltration, fibrin deposition, and a predominance of inflammatory cytokine production in the lung. In addition, a suppression of genes that encode proteins in antigen presentation pathways was observed which could have limited the ability of these animals to eventually mount a successful adaptive immune response to infection. These results provide a valuable insight into the host response to *H. parasuis *at the genomic level and identify genes that are candidates for involvement in disease resistance and susceptibility phenotypes. Further investigation is warranted into the molecular mechanisms governing these phenotypes and whether they operate in infections involving different strains of *H. parasuis *or swine of different genetic backgrounds.

## Methods

### Bacterial challenge and selection of pigs for array experiments

The tissue samples used for the experiments described in this paper were collected as part of a previous study [14]. Although the challenge protocol is described in more detail in that paper, it is summarized below to provide clarification on how animals were assigned to susceptibility categories. This information is relevant to the selection of pigs for microarray experiments. Briefly, six boars from a commercial line (a Landrace-Duroc synthetic) were mated with Landrace × Large White sows. The offspring were removed immediately after farrowing and deprived of maternal colostrum. They were disinfected and then raised in an isolation facility. Piglets at 21 days of age were inoculated intra-tracheally with the pathogenic 29755 strain of *H. parasuis *(serovar 5). Control animals were mock-inoculated with phosphate buffered saline (PBS). Approximately equal numbers of pigs were euthanized at 24, 48 and 72 hours post-inoculation. In total, 126 animals were inoculated with *H. parasuis *and 29 with PBS. Animals were inoculated in one of 10 independent challenge experiments. Approximately 4-5 inoculated pigs and 1 mock-inoculated control animal were harvested at each time-point in each experiment.

Animals were given a daily score of 0-3 based on the presence and severity of 4 clinical signs associated with Glässer's disease (demeanour, respiratory problems, lameness, and neurological signs). At necropsy, animals were scored 0-3 for the presence and severity of 9 Glässer's disease lesions (pleurisy, hydrothorax, pericarditis, hydropericardium, peritonitis, ascites, meningitis, arthritis, and pneumonia). Swabs were collected from 6 tissue sites (pleura, pericardium, peritoneum, carpal joint, foot joint, and meninges) and tested for *H. parasuis *by culture and PCR. Five mm^2 ^lung tissue samples were collected in RNA Later (Ambion) within 15 minutes of the animal being killed and stored at - 80 °C. Samples were selectively taken from areas of the lungs exhibiting Glässer's disease lesions where present.

Each animal inoculated with bacteria was classified into one of four categories for relative susceptibility to Glässer's disease: 'Fully Resistant' (FR), 'Less Resistant' (LR), 'Less Susceptible' (LS) and 'Fully Susceptible' (FS). Animals exhibiting extremes of susceptibility, together with mock-inoculated control animals, were chosen for microarray experiments. The 6 'resistant' animals chosen for expression profiling were all classified as 'Fully Resistant' (FR). 'FR' pigs were negative for *H. parasuis *by culture or PCR at all tissue sites, had lesions with a score of 1 in no more than two tissues, or a total lesions score of no more than 3 across 9 sites, and had clinical signs with a score of 1 in no more than two categories. The 'susceptible' animals were selected to match the 'FR' animals for sire and time-point. Because of these restrictions, it was not possible to select 6 'FS' animals: 4 'FS' and 2 'LS' animals were chosen and this group of 6 animals are referred to as 'Susceptible'. These animals had 4 or more sites positive for *H. parasuis *by culture or PCR, a total lesion score of at least 5 across 9 sites, and a total clinical signs score of 3 or more. Mock-inoculated 'Control' animals were negative for *H. parasuis *by culture or PCR at all tissue sites, and had no lesions or clinical signs associated with Glässer's disease. They were matched for sire with 'FR' and 'Susceptible' animals. It was not possible to use lung RNA from multiple control animals for the same sire and time-point in the pools as only one animal from each sire at each time-point was designated as a 'Control' animal in the original challenge experiments. Therefore pools were made from RNA from 'Control' animals of the same sire but different time-points (24, 48, and 72 hours). The animals used in the microarray experiments are described in more detail in Table 1. All animal procedures followed regulations of the European Directive 86/609/CEE.

### RNA extraction

RNA was extracted from lung tissue using Tri-Reagent (Sigma Aldrich). The RNA samples were further purified using an RNeasy mini kit (Qiagen) incorporating an on-column digestion of DNA using RNase-free DNAse I (Qiagen). RNA integrity was determined by electrophoresis on an Agilent 2100 Electrophoresis Bioanalyzer (Agilent Technologies). Only samples with a 28:18 S rRNA peak of > 1 were included in subsequent analyses.

### Detection of *H. parasuis *by RT-PCR

A 500 ng amount of total RNA from each sample in a 20 μl volume was reverse-transcribed using Superscript II reverse transcriptase (Invitrogen), and the remaining RNA digested using RNase H (Invitrogen). PCR was carried out using HotStar *Taq *polymerase (Qiagen) and primers designed to generate an amplicon of 821 bp from the 16 S rRNA gene of *H. parasuis *[19] using the following conditions: 95°C for 15 minutes, followed by 37 cycles of 94°C for 30 seconds, 59°C for 30 seconds, and 72°C for 150 seconds. A positive control PCR was carried out using primers for the pig β-actin gene (Forward primer: 5'-TCCCTGGAGAAGAGCTACGA-3'; Reverse primer: 5'-TGTTGGCGTAGAGGTCCTTC-3') generating an amplicon of 182 bp on cDNA and 280 bp on genomic DNA (the primers were designed either side of intron 4 of the pig gene). The reaction conditions were: 95°C for 15 minutes, followed by 32 cycles of 94°C for 30 seconds, 57°C for 30 seconds, and 72°C for 30 seconds. RT-PCRs were performed twice for each sample with each primer pair.

### Microarray labelling and hybridization protocols

Using a reference design, 'target' cDNA was prepared from individual 'FR' and 'Susceptible' pigs and co-hybridized to microarrays with reference cDNA made using lung RNA from pools of mock-inoculated control animals. Four technical replicates (2 sets of dye-swaps) were performed for each animal. The microarray used for this study was the Pig Genome v1.0 + Extension set oligonucleotide microarray (Operon). These arrays contained 13,297 70-mer probe sequences designed from The Institute for Genomic Research (TIGR) *Sus scrofa *Gene Index (SsGI) database release 5.0 (2002). The probes were printed in duplicate. Labelled cDNA targets were prepared using a two step process. First, the target population was amplified by RT-PCR using the SMART template switching system (BD Biosciences). Second, the PCR product was labelled with Cy3 or Cy5-dCTP (GE Healthcare) using a Bioprime DNA Labelling System (Invitrogen). A 500 ng amount of total RNA was mixed with 10 pmol of 3' SMART CDS primer IIA (5'-AAGCAGTGGTATCAACGCAGAGTAC-T_30_VN-3') and 10 pmol template switching primer [5'-d(AAGCAGTGGTATCAACGCAGAGTACGC)r(GGG)-3']. Reverse transcription was carried out using 'Powerscript' reverse transcriptase according to the manufacturer's instructions (BD Biosciences). PCR was performed using the first strand cDNA as template with the following PCR primer (5'-AAGAGT GGTATCAACGCAGAGT-3') and HotStar Taq (Qiagen). The conditions were: 95°C for 15 minutes, then 18 cycles of 94°C for 30 seconds, 65°C for 30 seconds and 68°C for 6 minutes. PCR products were purified using a MinElute PCR purification kit (Qiagen). Purified PCR product in a random primer mix was denatured at 95°C for 5 minutes. A low-dCTP mix (5 mM dATP, dGTP, and dTTP; 2 mM dCTP), Cy3 or Cy5-dCTP (1mM) (GE Healthcare) and 40 units of Klenow polymerase (Invitrogen) were added and the reactions incubated at 37°C for 2 hours. Labelled products were purified using Autoseq G-50 columns (GE Healthcare). Cy3 and Cy5-labelled target were combined with 4 μg of Porcine Hybloc (Applied Genetic Laboratories), 8 μg of poly dA oligonucleotide (Sigma-Genosys) and 4 μg of yeast tRNA (Sigma-Aldrich). The DNA was ethanol precipitated and resuspended in 50 μl OpArray Hyb Buffer (Operon) (final concentration of each dye was 0.8 pmol/μl). Microarrays were pre-hybridized in OpArray Pre-hyb buffer prior to hybridization. The labelled target DNA was denatured at 65°C for 5 minutes then applied to the array surface under a Lifterslip (Thomas Scientific). Hybridizations were performed at 42°C for 15 hours. Following hybridization, slides were washed using Operon wash buffers and dried by centrifugation. Slides were scanned using a GenePix 4100A Personal Scanner (Axon Technologies) and signal intensities quantified using Bluefuse 3.0 (Bluegnome). Features with irregularities affecting signal intensity were removed by manual flagging and mean signal intensities for duplicate spots were calculated.

### Microarray data analysis

Microarray data were analyzed using 'limma' (linear models for microarray analysis) [76]. Data were normalized using a global Loess algorithm. Only genes with data available from all hybridizations in the comparison of interest were used. A linear model was fitted to the log2 transformed data to estimate variability. For statistical analysis of differential gene expression, an empirical Bayes method was used to moderate the standard errors of the log-fold changes. A Benjamani-Hochberg (BH) correction was applied to the p values of the log fold changes to correct for multiple testing [77]. Genes were categorized as differentially expressed if they were ≥ 1.75 fold more or less highly expressed in one group compared to the other and had a BH adjusted p value of < 0.05. Sequences from differentially expressed genes with no sequence annotation were used to re-screen the non-redundant and EST Genbank databases for gene matches using the nucleotide BLAST algorithm [78]. The online software tool 'GoStat' was used to identify human Gene Ontology (GO) terms that were significantly over-represented in gene lists as compared to the genome as a whole, indicating their potential importance to the ascribed process [79].

### Quantitative RT-PCR (RT-qPCR)

Two-step quantitative RT-PCR was performed on the same total RNA samples used for the microarray experiments. In total, RT-qPCR was carried out for 23 differentially expressed genes and 3 reference genes. Primer sequences are listed in additional file 5. The forward primer of each pair was designed to span a predicted intron/exon splice junction in the pig gene (predicted by alignment with the human RefSeq sequence). In addition, the primers were designed to exons present in all reported splice variants of the human gene in the Entrez Gene database at NCBI. First, 1 μg of total RNA was reverse transcribed into first-strand cDNA using the Quantitect Reverse Transcription kit (Qiagen) according to the manufacturer's recommended protocol. Real-time PCR was carried out in triplicate using a 1/50 dilution of each RT reaction, and once using a 1/10 dilution of an RT negative control (an RT performed without reverse transcriptase). PCR assays were carried out using the Quantitect SYBR green PCR kit (Qiagen) with 0.3 μM of each primer (Sigma-Genosys) in a 10 μl volume. Reactions were run on a Rotorgene 3000 thermal cycler (Corbett Research Ltd) using the following amplification conditions: 95°C for 15 minutes, then 40 cycles of 94°C for 15 seconds, X °C for 30 seconds and 72°C for 30 seconds, where X is between 54 and 57°C depending on the primer pair used. A melt-curve analysis of each primer pair was carried out after PCR to verify the specificity of the PCR assay. Reaction efficiencies and quantification cycle (C_q_) values were calculated by the Rotorgene software using a standard curve derived from real-time RT-PCR carried out on a 10-fold dilution series of a standard consisting of RNA from 'FR', 'Susceptible', and 'Control' animals in equal proportions.

### Analysis of RT-qPCR data

For each gene of interest, the mean Cq value of the technical and biological replicates in the 2 groups being compared (S v C, FR v C, FR v S, or S v FR) were determined. The relative expression value (Q) was calculated using the delta Ct formula [80]. The values for Q and its standard error were then adjusted by a normalization factor calculated by determining the geometric mean of the relative expression ratio for 3 reference genes [81]: ribosomal large subunit protein 8 (*RPL8*), NADH dehydrogenase 1, beta subcomplex protein 10 (*NDUFB10*), and Huntingtin interacting protein 1 (*HIP1*). These genes were selected from the microarray results for their stability of expression across all samples (< 1.12 fold differentially expressed). The statistical significance of differential expression between the groups being compared was determined by non-parametric randomization tests using REST 2005 software [82].

## Authors' contributions

JMW carried out all lab work and data analysis as part of his PhD studentship. CAS contributed to the design of the microarray experiments and interpretation of microarray data. LG provided data from the study of Blanco *et al. *(2008) and represented the industry partner (PIC) in a supervisory role. AWT acted as academic supervisor and provided mentorship throughout the PhD studentship. All authors read and approved the final manuscript.

## Supplementary Material

Additional file 1 **Lists of DE genes from the FR v C, S v C, FR v S and S v FR gene expression comparisons at 24 hours post-inoculation**. The DE genes for each comparison are listed in separate tabs of this Microsoft Excel file. Data for DE transcripts representing the same gene are also listed in tabs. Annotation for each gene consists of the SsGI identifier (IDs start TC), the Genbank Reference sequence identifier and name of the best matched human gene, and the pig oligonucleotide identifier and sequence. The data consist of Log2 gene expression ratios and their associated variance and p values (both corrected and uncorrected for multiple testing), in addition to none log-transformed fold changes and amplitude of probe signal (signal intensity). Click here for file

Additional file 2 **Lists of DE genes from the FR v C, S v C, FR v S and S v FR gene expression comparisons at 72 hours post-inoculation**. The DE genes for each comparison are listed in separate tabs of this Microsoft Excel file. Data for DE transcripts representing the same gene are also listed in separate tabs. Annotation for each gene consists of the SsGI identifier (IDs start TC), the Genbank Reference sequence identifier and name of the best matched human gene, and the pig oligonucleotide identifier and sequence. The data consist of Log2 gene expression ratios and their associated variance and p values (both corrected and uncorrected for multiple testing), in addition to none log-transformed fold changes and amplitude of probe signal (signal intensity). Click here for file

Additional file 3 **Lists of DE genes from the expression profiles of 'Fully Resistant' animals at 24 and 72 hours post-inoculation**. The expression profiles of 'Fully Resistant' animals contain those genes that are differentially expressed in both the 'FR v C' and 'FR v S' comparisons. The expression profiles of 'Fully Resistant' animals at 24 and 72 hours post-inoculation are shown on separate tabs. Data for DE transcripts representing the same gene are also listed in separate tabs. Annotation for each gene consists of the SsGI identifier (IDs start TC), the Genbank Reference sequence identifier and name of the best matched human gene, and the pig oligonucleotide identifier and sequence. The data for each comparison consists of Log2 gene expression ratios and their associated variance and p values (both corrected and uncorrected for multiple testing), in addition to none log-transformed fold changes and amplitude of probe signal (signal intensity). Click here for file

Additional file 4 **Lists of DE genes from the expression profiles of 'Susceptible' animals at 24 and 72 hours post-inoculation**. The expression profiles of 'Susceptible' animals contain those genes that are differentially expressed in both the 'S v C' and 'S v FR' comparisons. The expression profiles of 'Susceptible' animals at 24 and 72 hours post-inoculation are shown on separate tabs. Data for DE transcripts representing the same gene are also listed in separate tabs. Annotation for each gene consists of the SsGI identifier (IDs start TC), the Genbank Reference sequence identifier and name of the best matched human gene, and the pig oligonucleotide identifier and sequence. The data for each comparison consists of Log2 gene expression ratios and their associated variance and p values (both corrected and uncorrected for multiple testing), in addition to none log-transformed fold changes and amplitude of probe signal (signal intensity). Click here for file

Additional file 5 **Primer sequences for RTqPCR**. Primers were designed from the pig expressed sequence tag (EST) sequence indicated. Forward primers were designed over intron/exon boundaries predicted from alignment of pig and human RefSeq sequence. Click here for file
