# Supplementary material for: Gene expression profiling in the lungs of pigs with different susceptibilities to Glässer's disease
Source: BMC Genomics. 2010 Jul 29;11:455. doi: 10.1186/1471-2164-11-455 (PMC3017779; doi:10.1186/1471-2164-11-455)
Supplement: Additional file 5 — Primer sequences for RTqPCR. Primers were designed from the pig expressed sequence tag (EST) sequence indicated. Forward primers were designed over intron/exon boundaries predicted from alignment of pig and human RefSeq sequence. [file 1471-2164-11-455-S5.DOC]

**Primer sequences for RTq-PCR.**

| **Gene symbol** | **Pig EST ID** | **Primer sequences (5′-3′)** | **Ta (°C)** | **Amplicon size (bp)** |
| --- | --- | --- | --- | --- |
| *ABCA6* | DN102373 | Forward: CATTTCACGATTGGTGGACTC  Reverse: ATCCAGAAGCAGGACAGGTG | 57 | 136 |
| *ALOX5AP* | EW080040 | Forward: TCCAGAACGCATTCTTTGC  Reverse: CACAGTTCTGGTTGGCAGTG | 55 | 120 |
| *APOD* | BP151360 | Forward: CAAGGAGTTGAGAGCTGACG  Reverse: GGACCCAGTATGGAGCTGAT | 55 | 128 |
| *CD163* | AJ311716 | Forward: TGCTCAGAAATTGCAAAGAGC  Reverse: TCGCTTCTGAGTCCAAATGAG | 54 | 120 |
| *COL1A1* | AK236626 | Forward: GGATTCCAGTTCGAGTACGG  Reverse: CGACGGATTCCAGTTCGAG | 57 | 102 |
| *COL1A2* | AJ666918 | Forward: GCTGGATCAGTGGGTGAAC  Reverse: CCATCACGACCAGCTTCAC | 57 | 122 |
| *C4BPA* | EW525377 | Forward: TGTCTCCCAAACCTGGAGGA  Reverse: GCTGCAAGGCATGTTTTTCC | 55 | 161 |
| *HIP1* | AW435804 | Forward: CAGAATCGAGGAGATGCTCAG  Reverse: TGCATGAGGCTGGTACAGG | 57 | 99 |
| *IGHA* | AK238269 | Forward: AAACCAGCCCCAAAATCTTC  Reverse: CAACAGACAGGTGCTTTCCAC | 55 | 120 |
| *IL1B* | AJ747049 | Forward: TGAAGAGAGAAGTGGTGTTCTGC  Reverse: GGTACAGATTCTTTCCCTTGATCC | 54 | 105 |
| *IL1RN* | CJ003758 | Forward: CCAGTTGGAGCCAGTTAACATC  Reverse: ACTGTCGGAGCGGATGAAG | 55 | 82 |
| *LPL* | DT322483 | Forward: CTGGACGGTGACAGGAATG  Reverse: TCCCGCAGATATTGGATAATG | 55 | 136 |
| *LTA4H* | CJ014141 | Forward: TCTACGCAGCCTGACTCTG  Reverse: GGGATCCTTTGTAGCTTTGTC | 55 | 110 |
| *LTBP4* | BX920434 | Forward: GAGACAGCCGAGTACCAGTC  Reverse: GGTCTCGGAAGAGCTGACAC | 57 | 109 |
| *LTF* | M81327 | Forward: CTGTCTTGGACAACACGAATG  Reverse: AGCCTCAGTCACAGGCTTC | 54 | 113 |
| *NDUFB10* | DN106278 | Forward: CCCTCGCGAGAGAGTTTATC  Reverse: TGTCCTTCTCCTCGCACTC | 57 | 108 |
| *OAS2* | BF199347 | Forward: CAAATGGTGTGAACGCAAAC  Reverse: CTTCTCCCAGGCATAGATGG | 57 | 88 |
| *RETN* | EW415102 | Forward: CGCCAGTTTCCTAATTCCTC  Reverse: CAGTGACAGCAAAGCCTGAG | 55 | 107 |
| *RPL8* | BP170733 | Forward: CATGAACCCTGTGGAGCATC  Reverse: CAGCAATGAGACCCACTTTCC | 57 | 110 |
| *SOD2* | BP159718 | Forward: GGAACCCAAAGGGGAATTG  Reverse: CTTGTTGAAACCGAGCCAAC | 57 | 130 |
| *HLA-DM* | BP157198 | Forward: ACCGGATGCCCTGTATCTC  Reverse: CAACAGACAGGTGCTTTCCAC | 57 | 125 |
| *TF* | BX921796 | Forward: AGGGGCTTTCAGGTGTCTG  Reverse: AGATCTTTTGCCCAATCATCC | 54 | 108 |
| *TGFBI* | CF364246 | Forward: GAACAAACTCTTGGGCAATG  Reverse: ACTTCCAGCTTGTCACCTTG | 56 | 132 |
| *TMSL3* | CX063675 | Forward: TTGGTGAAGGAGGAAACTGG  Reverse: TTACAGCCCTCAGGACATCAC | 57 | 80 |
| *TNC* | BP143771 | Forward: ACAGTCAGGGTGTCAACTGG  Reverse: GCTGGGTCTCAGCTTCATC | 57 | 80 |
| *TNFAIP6* | BI342110 | Forward: AACCCACATGCAAAAGAGTG  Reverse: CCAGTAGCAGATTTGGTTATCATC | 57 | 103 |
